# A toolbox for decoding BCI commands based on event-related potentials

**DOI:** 10.3389/fnhum.2024.1358809

**Published:** 2024-03-04

**Authors:** Christoph Reichert, Catherine M. Sweeney-Reed, Hermann Hinrichs, Stefan Dürschmid

**Affiliations:** ^1^Department of Behavioral Neurology, Leibniz Institute for Neurobiology, Magdeburg, Germany; ^2^Neurocybernetics and Rehabilitation, Department of Neurology, Otto von Guericke University, Magdeburg, Germany; ^3^Center for Behavioral Brain Sciences, Otto von Guericke University, Magdeburg, Germany; ^4^Department of Neurology, Otto von Guericke University, Magdeburg, Germany; ^5^Helen Wills Neuroscience Institute, University of California, Berkeley, Berkeley, CA, United States; ^6^Department of Cellular Neuroscience, Leibniz Institute for Neurobiology, Magdeburg, Germany

**Keywords:** canonical correlation analysis, BCI, ERP, P300, N2pc, speller

## Abstract

Commands in brain-computer interface (BCI) applications often rely on the decoding of event-related potentials (ERP). For instance, the P300 potential is frequently used as a marker of attention to an oddball event. Error-related potentials and the N2pc signal are further examples of ERPs used for BCI control. One challenge in decoding brain activity from the electroencephalogram (EEG) is the selection of the most suitable channels and appropriate features for a particular classification approach. Here we introduce a toolbox that enables ERP-based decoding using the full set of channels, while automatically extracting informative components from relevant channels. The strength of our approach is that it handles sequences of stimuli that encode multiple items using binary classification, such as target vs. nontarget events typically used in ERP-based spellers. We demonstrate examples of application scenarios and evaluate the performance of four openly available datasets: a P300-based matrix speller, a P300-based rapid serial visual presentation (RSVP) speller, a binary BCI based on the N2pc, and a dataset capturing error potentials. We show that our approach achieves performances comparable to those in the original papers, with the advantage that only conventional preprocessing is required by the user, while channel weighting and decoding algorithms are internally performed. Thus, we provide a tool to reliably decode ERPs for BCI use with minimal programming requirements.

## 1 Introduction

Brain-computer interfaces (BCIs) can be controlled using brain signals recorded with noninvasive techniques such as electroencephalography (EEG) and have a wide range of applications (Saha et al., 2021). Different EEG features can be used to distinguish between user intentions, including the sensory motor rhythms (SMR) (Yuan and He, 2014), steady-state visually evoked potentials (SSVEP) (Vialatte et al., 2010), or event-related potentials (ERPs) (Fazel-Rezai et al., 2012; Chavarriaga et al., 2014). ERPs are characterized by deflections in the EEG that are locked to a stimulus event. The most frequently applied ERP in BCIs is the P300 response, elicited by a rare and unpredictable sensory stimulus (target or oddball) embedded in a series of standard stimuli (nontarget). An oddball stimulus correctly recognized as the target results in a signal deflection 300–600 ms after stimulus onset in sensors around the vertex. However, achieving a reliable detection of brain signals, such as the P300, does not rely on a particular set of sensors consistent across tasks and participants. This has led to different approaches for channel selection, such as recursive channel elimination (Rakotomamonjy and Guigue, 2008), mutual information maximization (Lan et al., 2005) and methods based on genetic algorithms and artificial neural networks (Yang et al., 2012). An alternative to channel selection is the linear transformation of the sensor space into a new surrogate sensor space, in which a reduced number of sensors represent the informative signal with reduced noise (Cohen, 2017). The advantage of such a spatial filter approach is that the channel weights are determined from training data and sensor selection is implicitly performed by down-weighting irrelevant channels. As a result, relevant information for signal classification is retained from all channels, and individual participant variation or differences in electrode placement do not result in loss of relevant information. The computational effort of this approach is lower than repeatedly training and testing a classifier for recursive feature elimination or for applying genetic algorithms and is thus better suited to BCI use. Application of such linear transformation methods for signal classification includes the common spatial pattern (CSP) approach, a well-established spatial filter for decoding SMRs (Blankertz et al., 2008; Arpaia et al., 2022), and canonical correlation analysis (CCA) for decoding SSVEPs (Lin et al., 2007). SMR and SSVEPs are oscillatory signals, which are characterized by frequency, amplitude and phase information, whereas feature extraction for BCIs is commonly restricted to amplitudes, neglecting the phase information. ERPs are time-locked signals and therefore feature extraction methods used for ongoing oscillations, including SSVEP, are not suitable. Nevertheless, CCA can be applied to ERP decoding using a suitable model as a reference function and result in enhanced decoding accuracies (Spüler et al., 2014). Including CCA has also proved advantageous in decoding P300 signals from the magnetoencephalogram (MEG) (Reichert et al., 2016, 2017) as well as in decoding the N2pc for various EEG-based BCI applications (Reichert et al., 2020a,b, 2022).

Open source toolboxes can make it easier for scientists to implement established algorithms. Several general-purpose toolboxes, which implement or provide an interface to several algorithms, are available for processing EEG/MEG data (Delorme and Makeig, 2004; Oostenveld et al., 2011) or for performing benchmark tests using open BCI datasets (Jayaram and Barachant, 2018). Here we extend the variety of publicly available algorithm implementations and introduce the ERPCCA toolbox^1^, which implements CCA as a tool for extracting canonical ERPs. The strength of the toolbox is the detection of a user’s intention that results from a series of stimuli, such as in P300-based speller applications (Rezeika et al., 2018; Maslova et al., 2023). Likewise, the ERPCCA toolbox is suitable for binary single event decoding such as the detection of error-related potentials. It is thus most suitable for scientists whose intention is to implement a BCI based on ERPs reflecting attention to sensory stimuli, but it is also useful for offline investigation of BCI data sets and ERPs reflecting two conditions. In this paper, we demonstrate the capabilities of the toolbox using four openly available datasets and compare the decoding accuracies of different classifier and feature set combinations.

## 2 Materials and methods

### 2.1 Decoding approach

#### 2.1.1 Analyzing brain signals using CCA

Canonical correlation analysis (CCA) (Hotelling, 1992) is a statistical approach for maximizing the correlation of two sets of canonical variates *U* and *V* by determining the matrices *A* and *B*, that linearly transform two sets of random vectors, denoted in matrix form as *X* and *Y*:


(1) $$\left(U,V\right)={\text{argmax}}_{A,B}\,\text{corr}\,\left(X\,A,Y\,B\right)$$

An implementation that solves this optimization problem is available in the MATLAB^®^ Statistics and Machine Learning Toolbox™ (*canoncorr*), which is required for using the ERPCCA toolbox. We apply CCA to a set of training data where we assign *X* as a matrix of brain signals of size *n* × *d*_1_ and *Y* as a matrix of model signals of size *n* × *d*_2_, where *n* is the number of sample points, *d*_1_ is the number of sensors or electrodes comprising the brain signals, and *d*_2_ is the number of model signals. This results in the canonical coefficients *A* of size *d*_1_ × *d* that linearly weight the brain signals and serve as a spatial filter, and *B* of size *d*_2_ × *d* that linearly weight the model signals and reflect the contribution of a model signal to a specific component. The number of components *d* depends on *d*_1_ and *d*_2_ and is the minimum of the ranks of *X* and *Y*. Furthermore, the components *i* = 1⋅⋅⋅d are sorted by their correlation ρ_*i*_(*u*_*i*_, *v*_*i*_) in descending order. Both *X* and *Y* are concatenations of epochs. An epoch is an interval following a single event, for example, a visual stimulus, and comprises multiple time-varying signals, for example, EEG signals. While *X* is a concatenation of individual epochs of electrophysiological recordings, *Y* is a concatenation of template epochs that are repeated according to their condition, for instance, target and nontarget events.

However, the definition of model signals is a challenge, since the ground truth ERPs are unknown, especially for an individual person performing a specific task. As the assumption is that the stimulus signal is directly reflected in the brain response, a common approach in SSVEP BCIs is to model *Y* using sine and cosine functions of the same frequency as the flicker stimuli and their harmonics. In a study investigating the stimulus-response mapping of continuous auditory stimuli, the authors used the envelope of the auditory stimulus as the model signal (de Cheveigné et al., 2018). Here, however, the focus is on ERPs, which reflect higher cognitive functions such as attention, and not only the sensory input. One approach is to use the average ERP signal as the model signal (Spüler et al., 2014). The advantage is that noise-reduced individual brain responses are directly used as the model signal. Our approach in previous work was to use identity matrices of size *m* × *m*, where *m* is equal to the sample points in a single epoch (Reichert et al., 2016). The advantage of this approach is that the canonical components in *V* are not limited to linear combinations of defined time courses, as with average ERPs, but they can result in arbitrary time courses, because each model signal can be considered an impulse function responsive to one sample point in the epoch, and the canonical coefficients directly mirror the time course of the optimal model signals, since *I*_*m*_*B* = *B*. In the ERPCCA toolbox, we implement both approaches, using average signals and impulse functions as model signals, which can be selected as an option.

#### 2.1.2 Using the learned model

Once we have determined the canonical coefficients in matrices *A* and *B* from the concatenated set of training data, we can use these filters to extract features from new, unseen test data. Commonly, only a few components are informative for discriminating event-related brain responses. Therefore, only the *k* first components in *A* and *B* are used to extract the features. The component limit is an option in the toolbox that can be set to a fixed number *k* or to a *p*-value resulting in selecting the most significant components as obtained from statistics available from the *canoncorr* function. A single trial can be composed of a sequence of epochs, e.g., epochs following target and nontarget events to identify one letter for a speller. The test data are transformed to a matrix $\hat{X}$ of size $\hat{n}\times d_{1}$ where $\hat{n}$ is the number of sample points after concatenating the epochs in a sequence. Furthermore, for each class *c* (e.g., spellable character), a sequence of model signals ${\hat{Y}}_{c}$ of size $\hat{n}\times d_{2}$ is generated. ${\hat{Y}}_{c}$ can be composed of only model signals related to target events or involve the nontarget events as well. In the latter case, the model signals determined from nontarget events are multiplied by −1, which leads to optimizing the spatial filter subject to the difference wave of target and nontarget epochs. Consequently, the common signal (usually the sensory input) is subtracted and only the difference is preserved (the intention, generated by cognitive processing).

#### 2.1.3 Meaning of canonical coefficients

To interpret the estimated canonical components with respect to underlying cognitive processes, we take a closer look at the linear weight matrices *A* and *B*. The canonical coefficients in *A* serve as spatial filters, since they linearly combine the channels in *X*. However, high values in *A* do not necessarily reflect a high resemblance of these channel signals with the respective canonical variate. Instead, we can calculate the inverse of *A*, which can be used to transform the backward model *U* = *XA* (see Equation 1) to the forward model *X* = *U*(*A*^−1^)*T* + ϵ (Haufe et al., 2014). *A*^−1^ is referred to as the activation pattern, or simply the pattern, as it characterizes the spatial pattern of the activity in the components in *U* (Blankertz et al., 2008). We will refer to an activity pattern of the *j*th ranked canonical component as 𝕒_*j*_, where 𝕒_*j*_ is the *j*th column vector in (*A*^−1^)*^T^*.

Since the canonical coefficients in *B* are used to linearly combine the model signals, their meaning depends on *Y*. In the case of setting the model signal to the average across epochs, *B* again can be considered a spatial filter. In contrast, impulse model signals enable the direct interpretation of *B* as comprising component time courses. Each column in *I* corresponds to one sampling point in an epoch. When two conditions are involved, such as target and nontarget events, the columns in *B* signify event-related canonical difference waves. Conversely, when only one condition is involved, the columns in *B* represent event-related canonical potentials. We will therefore refer to the *j*th ranked canonical difference wave, obtained by using impulse model functions and involving two conditions, as *b_j_*, where *b_j_* is the *j*th column vector in *B*.

#### 2.1.4 Feature spaces and classifiers

We implemented two kinds of feature space. The first feature space is composed of the Pearson correlation coefficients of the canonical components, i.e., the columns in $\hat{U}=\hat{X}\,A$ and ${\hat{V}}_{c}={\hat{Y}}_{c}\,B$. We refer to this *k*-dimensional feature space as R. The second feature space is composed of the canonical variate $\hat{U}$ by concatenation of the time series of each component, resulting in *k*⋅*m* features. If one trial is composed of several repetitions, canonical epochs that correspond to the event of the currently considered class, e.g., the stimulus of a letter in a spelling paradigm, are averaged. We refer to this feature space as U.

Finally, to decode the brain responses, different classifier approaches may be used to determine the user’s intention based on the extracted features. We implemented four types of classifiers in the toolbox: support vector machine (SVM), linear discriminant analysis (LDA), and naïve Bayes classifier (nB), which are based on the implementations in the Statistics and Machine Learning Toolbox™, as well as an approach which is based on maximum correlation values and was first presented for CCA-based ERP decoding in Reichert et al. (2015). Hyper parameters that have an impact on the classification result, are internally estimated from the training data. For the SVM’s box constraint parameter *C*, we used the approach suggested by Joachims (2002),^2^ and for the LDA regularization parameter, we set the default value as implemented in the MVPA-light toolbox^3^ (Treder, 2020). Alternatively, using Matlab’s concept of function handles, arbitrary classifiers, including the definition of their parameters, can be applied. The ability to specify function handles also opens up the possibility of programming custom classifier functions and interfaces to other machine learning toolboxes.

Once a classifier is trained using a training dataset, a user’s intention can be predicted from test data. In this toolbox, ERP decoding relies on binary classification. The intended class is determined by identifying the class with the highest probability of exhibiting responses to target events. This probability is obtained through the Posterior Probability generated by the *predict* function of SVM, LDA, and nB. For the maximum correlation classifier, this probability is linked to the correlation coefficient in the R feature space. Consequently, the class is directly determined by identifying the maximum of averaged correlation coefficients across the *k* components. This classifier is only applicable to the R feature set. In contrast, SVM, LDA and nB are applicable to both R and U feature sets.

### 2.2 General usage of the toolbox and data preparation

**FIGURE 1:**
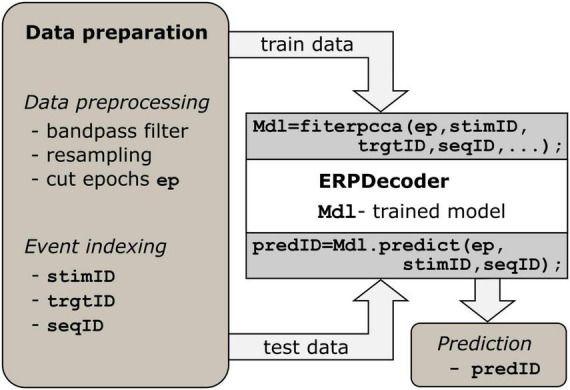
Processing steps. Data preparation is a prerequisite. Epoch data should have three dimensions (number of channels, number of samples and number of events). For each event, identifiers indexing the current stimulus, target, and trial/sequence are required. The *ERPDecoder* concatenates the epochs and model signals accordingly, and performs CCA and classifier training. Arrows indicate the flow of input and output data.

We implemented the algorithm described above such that users can apply it on a low level. Essentially, only two functions must be called to perform the decoding: *fiterpcca*, which returns a trained *ERPDecoder* object, and the *predict* function, which is applied to that object. Furthermore, we provide a function *crossvalerpcca*, which performs cross-validation. Below we describe how to prepare the data to successfully apply the toolbox, which is I–Data preprocessing and II–Event indexing. See Figure 1, as well as the examples in the repository^1^ for reference.

I—Data preprocessing. The brain data must be presented as a three-dimensional array of size [number of channels × number of samples × number of events]. Each epoch refers to the brain response to a single event. To achieve good results, we recommend bandpass filtering the data between 1.0 and 12.5 Hz to remove slow signal drifts and irrelevant fast fluctuations. If one trial consists of several events, as in speller paradigms, the trial might be filtered before final epoching to prevent edge effects. To reduce computational load, we suggest resampling the data to around 50 Hz.

II—Event indexing. Since stimulus information is essential for training the decoder, three vectors providing details of events are required. The stimulus identifier (*stimID*) denotes the stimulus that was presented, i.e., a number ranging from 1 to *c* if *c* classes were available, and is provided as a column vector. If multiple events occurred simultaneously, e.g., in a matrix speller where several characters are highlighted simultaneously, *stimID* can instead be specified as a matrix with one column per simultaneous event. In another column vector, the target stimulus identifier (*trgtID*) is required to indicate which of the *c* classes was the target class in that trial/sequence, i.e., all epochs that correspond to one trial/sequence should have the same value in *trgtID*. Finally, one column vector is required, which indicates a sequence identifier (*seqID*), i.e., all epochs that correspond to one trial/sequence of stimuli, should have identical values in *seqID*. The identifiers 1⋅⋅⋅*s* are recommended, where *s* is the number sequences.

Options that define which model signal, feature space, classifier, etc., is used can be assigned as name-value pairs. These values depend on the task at hand and can have a substantial impact on the results. One useful option is to define an alphabet which assigns characters to the classes 1⋅⋅⋅*c*. This results in readable predictions, e.g., spelled words rather than a series of stimulus identifiers. Another potentially useful option is the possibility of enabling/disabling the involvement of non-target events in the CCA. If the contrast parameter is set to 0, only target events are involved in the estimation of spatial filters. Otherwise, non-targets are involved as well, and model signals for non-targets are calculated as described in 2.1, resulting in contrasting the conditions. Note that this applies only to the CCA step. For classification, the spatial filters are applied to all epochs. All parameters and options are described in the help text of a function and in the documentation of the ERPCCA repository^1^.

### 2.3 Application to open datasets

#### 2.3.1 Dataset I—Matrix speller

The first dataset can be accessed on the BNCI Horizon 2020 webpage^4^ under the name “12. Visual P300 speller (003-2015).” It includes 10 subjects who participated in a short matrix speller session. The dataset provides 8 channels of EEG recordings and 10 trials per subject, a five-character word for training and another for testing. Each of the 36 characters was highlighted 15 times per trial. More details about the experiments, including the preprocessing, which was performed analogously here, can be found in the corresponding publication (Guger et al., 2009). Specifically, we applied a 4th order Butterworth band pass filter (0.5–30 Hz), performed baseline correction using the 116 ms interval before stimulus onset as the baseline, sampled the data to a 64 Hz sampling rate, and set the analysis window to 0–800 ms after stimulus onset. We performed 10-fold cross-validation involving all available trials and using different feature/classifier combinations. In contrast to the maximum correlation classifier and naïve Bayes classification, LDA and SVM are suitable for classifying data in high dimensional feature spaces. Consequently, we used feature/classifier combinations R/max, R/nB, R/SVM, R/LDA, U/SVM and U/LDA. We kept only three canonical components for the feature space calculations. Furthermore, we investigated the difference between using the impulse and the average model functions and also the impact of involving non-target epochs or not. Finally, we determined the decoding accuracy as a function of stimulus repetitions (5, 10 and 15 stimuli per trial) and performed a simulation of online sessions using the same training and test sets as during the BCI sessions.

#### 2.3.2 Dataset II—RSVP speller

Similarly to the first dataset, the second dataset was also recorded during spelling and is based on oddball events inducing a P300 response. However, it is gaze-independent since the stimuli were presented focally. It is available from the BNCI Horizon 2020 webpage (see text footnote 4) under the name “19. RSVP speller (010-2015).” The study, which uses RSVP to perform the BCI spelling task, involved 12 subjects equipped with 63 electrodes. Each of the 30 characters was presented 10 times per trial. Only sessions with 83 ms stimulus onset asynchrony (SOA) are available in the dataset although data with longer SOA were also recorded according to the corresponding publication, where classifier features were determined from averages within predefined time intervals (Acqualagna and Blankertz, 2013). Here we performed the preprocessing as follows. We applied a 4th order Butterworth band pass filter (0.01–20 Hz), performed baseline correction using the 116 ms interval before stimulus onset as baseline, downsampled the data to a 50 Hz sampling rate and set the analysis window to 0–800 ms after stimulus onset. We performed 10-fold cross-validation using the same feature/classifier combinations, model signals, and parameters as we did with dataset I. Finally, we determined the decoding accuracy as a function of stimulus repetitions (2, 4, 6, 8 and 10 stimuli per trial) and performed a simulation of online sessions using the same training and test sets as during the BCI sessions.

#### 2.3.3 Dataset III—Visual spatial attention

The third dataset^5^ includes 14 subjects who responded as to whether an auditorily presented number was even or not by directing their attention to a target color, associated with the intended response. Both colors, red and green, were presented 10 times per trial in a random order, one of them in either visual hemifield. In total, 144 trials per subject are available, including EEG from 12 occipital electrodes as well as the vertical and horizontal electrooculogram. Conceptually, the dataset differs from the first two datasets in how ERPs are extracted and how target detection is achieved. The main difference is that the target is present in each stimulus event, such that ERPs differ only with regard to spatial information pertaining to the target location rather than in the presence of a target. Consequently, with this dataset it only makes sense to contrast the conditions and set the contrast parameter accordingly. This is in line with what is typically done with this kind of data, which is to consider the difference wave of contralaterally vs. ipsilaterally presented targets. Similarly to the approach in the corresponding publication (Reichert et al., 2022), we filtered the data between 1.0 and 12.5 Hz, downsampled them to 50 Hz and considered 0–750 ms after onset as the analysis interval. We performed 10-fold cross-validation using the same feature/classifier combinations and model signals as we did with dataset I but did not vary the contrast parameter for the reason explained above. Finally, we determined the decoding accuracy as a function of stimulus repetitions (2, 4, 6, 8 and 10 stimuli per trial) and performed a simulation of online sessions using the same training and test sets as during the BCI sessions.

#### 2.3.4 Dataset IV—Error monitoring

Finally, we analyzed a dataset available from the BNCI Horizon 2020 webpage (see text footnote 4) under the name “22. Monitoring error-related potentials (013-2015).” The paradigm was designed to evoke error-related potentials following simulated BCI feedback and comprises 64-channel EEG data recorded from six participants, each of whom performed two sessions on different days with varying time differences between the recording sessions. According to the corresponding publication (Chavarriaga and Millán, 2010), erroneous feedback was provided in 40 and 20% of trials, but the openly available dataset comprises only blocks with 20% error probability and on average 536 (σ = 31) trials per session. Conceptually, the dataset differs from the datasets described above in that no sequence is available to enable benefit from repetitions of the same event to improve the signal-to-noise ratio, and that there is no target/nontarget associated with events that occur at different locations or different points in time. Our toolbox is designed to deal with this kind of binary single trial detection as well. In this case, the input variable *trgtID* is to be set as empty and *stimID* equals the classifier labels, which we set to 1 for error trials and to 2 for correct trials. We filtered the data between 1.0 and 10.0 Hz, in line with the original study, downsampled the data to a 32 Hz sampling rate, and considered 0–600 ms after onset as the analysis interval, after subtracting the baseline of the interval 250 ms before stimulus onset. We performed 10-fold cross-validation using the same feature/classifier combinations and model signals as we did with dataset I. Finally, we performed a simulation of online sessions using the same training and test sets as employed in the corresponding publication. Note that this was an offline analysis investigating the across-session accuracy of a classifier detecting error-related potentials with potential application to BCI.

## 3 Results

### 3.1 Dataset I—Matrix speller

**FIGURE 2:**
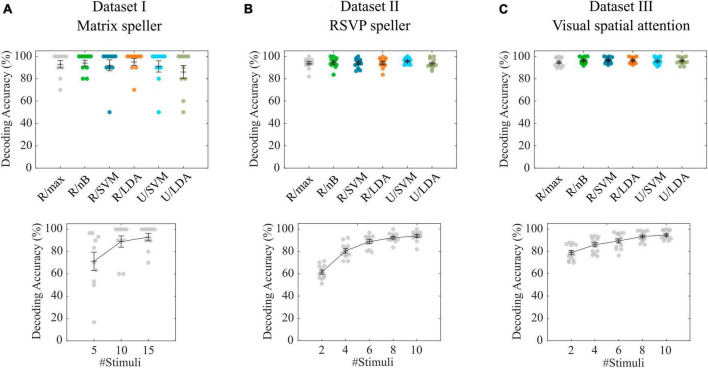
Cross-validation results for datasets I-III. **(A–C)** Decoding accuracies obtained with different feature space/classifier combinations (upper panels) and with the R/max approach involving different numbers of stimuli per trial (lower panels). Colored circles indicate single subject performances. Error bars show the mean accuracies and the standard error of the mean.

With the P300-based matrix speller data (Guger et al., 2009), the cross-validation of the tested combinations of feature spaces revealed an average decoding accuracy of 93.0% (σ = 10.6%) for the maximum correlation classifier (R/max), while the highest accuracy was achieved with R/LDA (μ = 95.0%, σ = 9.7%). All accuracies are shown in Figure 2A. Pairwise Wilcoxon signed-rank tests showed that none of the feature space/classifier combinations were significantly different from each other (*p* > 0.05). Figure 2A also shows the results of using only 5 and 10 stimulus repetitions compared with the full number of 15 repetitions using the R/max approach. A Wilcoxon signed-rank test revealed that 10 repetitions still achieve high accuracies (μ = 89.0%, σ = 16.0%), not statistically significantly different from using 15 repetitions (μ = 93.0%, σ = 10.6%). In contrast, involving only 5 repetitions significantly decreases the accuracy (μ = 71.3 %, σ = 26.0%). We repeated the analyses using the R/max approach and using average model functions instead of impulse model functions and found no differences in accuracies. Furthermore, we involved the non-target events by setting the contrast parameter accordingly, which did not result in significantly different decoding accuracies. Finally, we tested the R/max approach in a simulation, which uses the same training and test data as in the original experiment. This analysis resulted in an average decoding accuracy of 90.0% (σ = 19.4%). Note that only 5 characters per subject were available in either subset, training set and testing set. Online accuracies achieved during BCI control, which could potentially serve as a benchmark, were not documented in the dataset or elsewhere.

### 3.2 Dataset II—RSVP speller

With the P300-based RSVP speller data (Acqualagna and Blankertz, 2013), the cross-validation of the tested combinations of feature spaces revealed an average decoding accuracy of 93.8% (σ = 4.6%) for the maximum correlation classifier (R/max) while the highest accuracy was achieved with U/SVM (μ = 95.6%, σ = 2.7%). All accuracies are shown in Figure 2B. Pairwise Wilcoxon signed-rank tests showed that none of the feature space/classifier combinations were significantly different from each other (*p* > 0.05). Figure 2B also shows the results of using only 2, 4, 6 and 8 stimulus repetitions compared with the full number of 10 repetitions using the R/max approach. A Wilcoxon signed-rank test revealed that 8 repetitions still achieve high accuracies (μ = 92.3%, σ = 4.1%), not statistically significantly different from using 10 repetitions (μ = 93.8%, σ = 4.6%). In contrast, involving only 2, 4 and 6 repetitions significantly decreases the accuracy (μ = 61.5%, σ = 6.3%; μ = 80.6%, σ = 6.8% and μ = 88.8%, σ = 5.8%). We repeated the analyses using the R/max approach and using average model functions instead of impulse model functions and found no differences in accuracies. Furthermore, we involved the non-target events by setting the contrast parameter accordingly, which resulted in significantly higher decoding accuracies when using impulse model functions (μ = 96.4%, σ = 2.5%) and significantly lower decoding accuracies when using average model functions (μ = 80.9%, σ = 19.8%). Finally, we tested R/max in a simulation, which uses the same training and test data as in the original experiment. This analysis resulted in an average decoding accuracy of 93.8% (σ = 5.4%). Average online accuracy achieved during BCI control, was 93.6% for the open dataset (including only Color 83 ms blocks) according to the corresponding publication.

### 3.3 Dataset III—Visual spatial attention

In contrast to the previous two datasets, where targets and non-targets were presented at different time points, in the visual spatial attention (VSA) task, targets and non-targets were presented at the same time but in different visual hemifields (Reichert et al., 2022). Consequently, the position of a target can only be detected in the difference wave of left vs. right presented items, which can be modeled by setting the contrast parameter accordingly (a target-only model is not applicable). The cross-validation of the tested combinations of feature spaces revealed an average decoding accuracy of 94.7% (σ = 3.8%) for the maximum correlation classifier (R/max), while R/nB (μ = 96.2%, σ = 2.9%), R/SVM (μ = 96.5%, σ = 2.7%) and R/LDA (μ = 96.3%, σ = 2.7%) revealed significantly higher accuracies (*p* < 0.05). Figure 2C shows all accuracies achieved with feature space/classifier approaches as well as the results of using only 2, 4, 6 and 8 stimulus repetitions compared with the full number of 10 repetitions using the R/max approach. A Wilcoxon signed-rank test revealed that 10 repetitions achieve significantly higher accuracies than using less than 10 repetitions. We repeated the analyses using the R/max approach and using average model functions instead of impulse model functions and found a strong decrease (μ = 63.7%, σ = 16.0%), similar to that which we found analyzing the RSVP task. Finally, we tested R/max in a simulation, which uses the same training and test data as in the original experiment. This analysis resulted in an average decoding accuracy of 93.9% (σ = 5.6%). Average online accuracy achieved during BCI control was 90.1% (σ = 6.0%) for the open dataset according to the corresponding publication.

### 3.4 Dataset IV—Error monitoring

**FIGURE 3:**
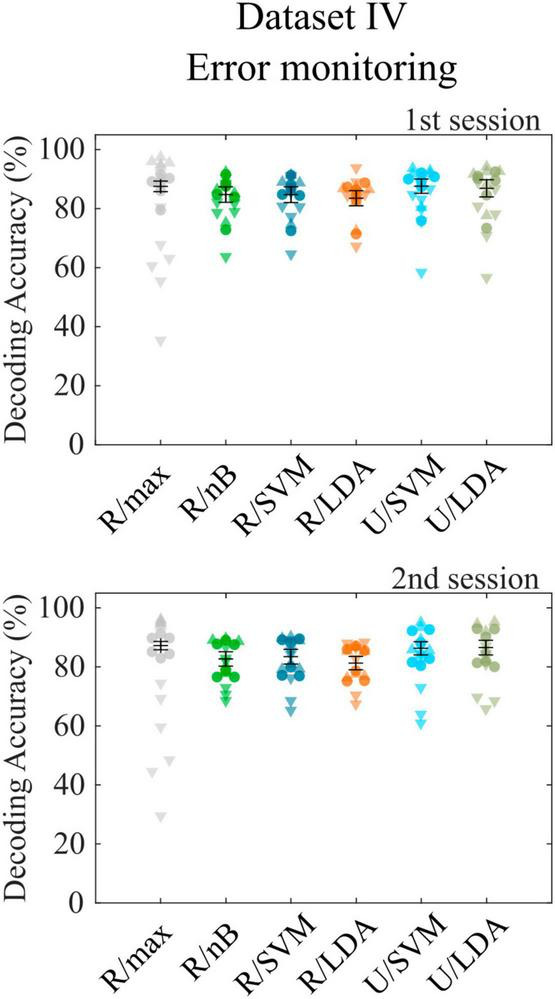
Cross-validation results for dataset IV. Decoding accuracies obtained with different feature space/classifier combinations using dataset IV for the first (upper panel) and second (lower panel) recording session. Colored circles indicate single subject performances, downward triangles and upward triangles indicate the accuracy for error trials and correct trials, respectively. None of the accuracies achieved with feature/classifier combinations differed significantly between recording sessions 1 and 2. Error bars show the mean accuracies and the standard error of the mean.

The final dataset (Chavarriaga and Millán, 2010) contains error-related potentials, which can only be decoded following a single event. The toolbox is designed not only to decode sequences of target and nontarget events but also accepts binary decoding tasks. Since the class sizes of the dataset are extremely imbalanced (20% error potentials), we report accuracies separately for both classes, as was done in the corresponding publication, to consider potential overfitting to the larger class. We observed a bias toward the larger class with the R/max approach, which revealed a total cross-validation accuracy of 87.4% (σ = 3.7%), while error trials contributed 57.4% accuracy (σ = 15.5%) and correct trials 94.9% accuracy (σ = 1.4%). All other approaches showed smaller biases toward the larger class. Highest decoding accuracies were achieved using the U/LDA approach (μ = 76.6%, σ = 10.0% for error trials; μ = 89.3%, σ = 5.4% for correct trials). Figure 3 shows all accuracies achieved with different feature space/classifier approaches. We repeated the analyses using average model functions instead of impulse model functions and found a significant accuracy decrease (μ = 73.2%, σ = 8.5% for error trials; μ = 86.0%, σ = 5.9% for correct trials) similar to the RSVP and VSA tasks. Both approaches outperformed the method introduced in the corresponding publication, where according to reconstruction from the figure showing within-subject cross-validation results [Figure 3 in Chavarriaga and Millán (2010)], the accuracies were around 60.0% for error trials and 77.0% for correct trials on average. Furthermore, we included only error trials in the CCA-based feature extraction by setting the contrast parameter accordingly, which resulted in not significantly different decoding accuracies. Finally, we tested U/LDA using impulse model functions in a simulation, which uses the same training and test data as in the original experiment. Note that this was a cross-session approach, where the second session, which was recorded many days later, was used as the test set. This analysis resulted in an average decoding accuracy of μ = 66.2% (σ = 15.8%) for error trials and μ = 74.1% (σ = 31.1%) for correct trials. Standard deviation is high, because subject 4 showed a bias toward correct trials and subject 6 showed a bias toward the error trials. Nevertheless, the average accuracy is comparable to that of the corresponding publication (μ = 63.2%, σ = 9.1% for error trials; μ = 75.8%, σ = 6.8% for correct trials).

The classification results of all parameter combinations for all analyzed data sets are summarized in Supplementary Table 1.

### 3.5 Exploring the canonical coefficients

For the two P300 datasets, we tested both involvement of only target events and also involvement of target and nontarget events in performing CCA. When we involved only the target events using the average model signals, in all cases *A* perfectly correlated with *B* (ρ = 1), where *A* only differed by a constant factor from *B*. This can be explained by the fact that the average was calculated from the same data in *X* that were combined to correlate with *YB*. Furthermore, when using only target events and the average across epochs as the model signal, the canonical coefficients in *A* perfectly correlated with those when using only target events and impulse model signals, i.e., the same spatial filter was determined although model signals differed. This can also be explained mathematically, because multiplication of any epoch with *I* does not change the epoch. Multiplying the concatenated brain signals in *X* with a concatenation of *I* is therefore equivalent to summing all target epochs, which is correlated with the average across target epochs. These properties are not valid when we involve the nontarget epochs, which have negated model signals to calculate difference waves, i.e., to contrast the conditions. In this case, *A* showed a high correlation with *B* (ρ = 0.94) when utilizing average model signals in the matrix speller dataset but a very low correlation (ρ = −0.02) in the RSVP dataset. This suggests that the ERPs in nontarget epochs are lower compared to the target epochs in the matrix speller but not in the RSVP speller. This is supported by comparing *A* when using average model signals with *A* when using impulse model signals, resulting in ρ > 0.99 for the matrix speller and ρ = 0.38 for the RSVP speller.

**FIGURE 4:**
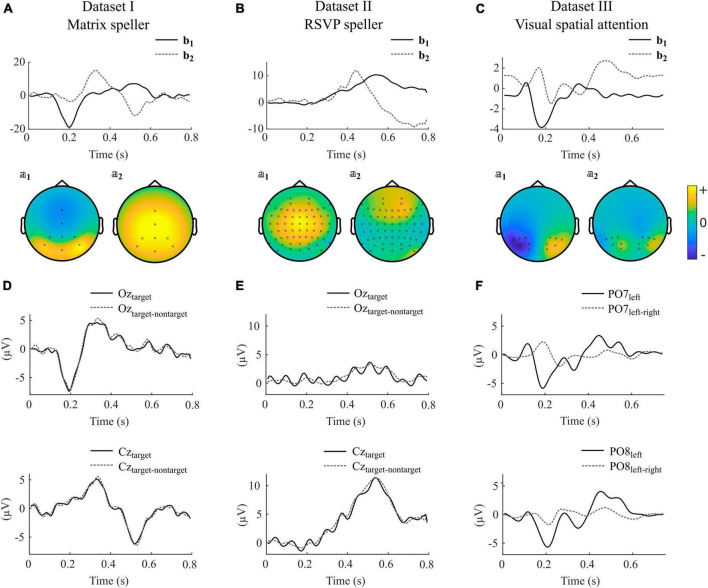
Selected canonical components as obtained with impulse model functions for datasets I-III. **(A–C)** Model signal coefficients (upper panels) and the corresponding activation patterns (second row) of an individual participant selected from datasets I-III. Units of *b_j_* and 𝕒_*j*_ are arbitrary. Positive values in 𝕒_*j*_ indicate similar shape in that channel as in *b_j_*, negative values indicate a reversed shape. **(D–F)** Average signals and difference waves of selected electrodes. The signals correspond to the same participants as shown in **(A–C)**. The electrodes are selected to show the ERPs that are commonly thought to control the BCI. The shown components for datasets I-III were taken from participants *s2*, *VPgcf*, and *P14*.

In Figure 4, we show selected components obtained by using impulse model signals as well as averaged ERPs and difference waves of specific electrodes of individual participants for each dataset. Note that the components are composed of different electrodes, influenced by several sources, and can therefore strongly vary between subjects in terms of their shape, their ranking, and their orientation.

Figure 4A, D illustrate that in the matrix speller, the first canonical component *b*_1_ resembles activity at electrode Oz, and its activity pattern 𝕒_1_ shows highest activity in occipital regions. This indicates that the sensory input, i.e., the foveal stimulation, is the main driving component. The P300 component is reflected in the second component, *b*_2_, and resembles activity at Cz. The difference wave at Oz resembles the ERP, indicating that nontarget activity is low compared to target activity. This is in line with the findings that canonical coefficients and decoding accuracies were not different with involvement of nontarget epochs in this dataset, indicating that the nontarget epochs have almost no impact on CCA-based feature extraction.

In contrast, Figure 4B, E indicate that in the RSVP speller, the highest ranked component is related to a P300-like response as reflected in *b*_1_, resembling activity at Cz, and its activity pattern 𝕒_1_. Importantly, Oz shows not the early negative ERP followed by the target stimulus but, specifically visible when not subtracting the nontarget epochs, a steady-state visually evoked potential induced by each single foveal stimulus (83 ms SOA).

In the VSA experiment, no nontarget events are present but the target switches between the left and right periphery. Figure 4C shows that the first canonical component *b*_1_ resembles the difference wave (target left-target right) at PO7 and in a reversed shape at PO8 (Figure 4F). The negative deflection 200 ms after target presentation contralateral to the respective electrode location is typically found in visual search experiments and known as the N2pc.

**FIGURE 5:**
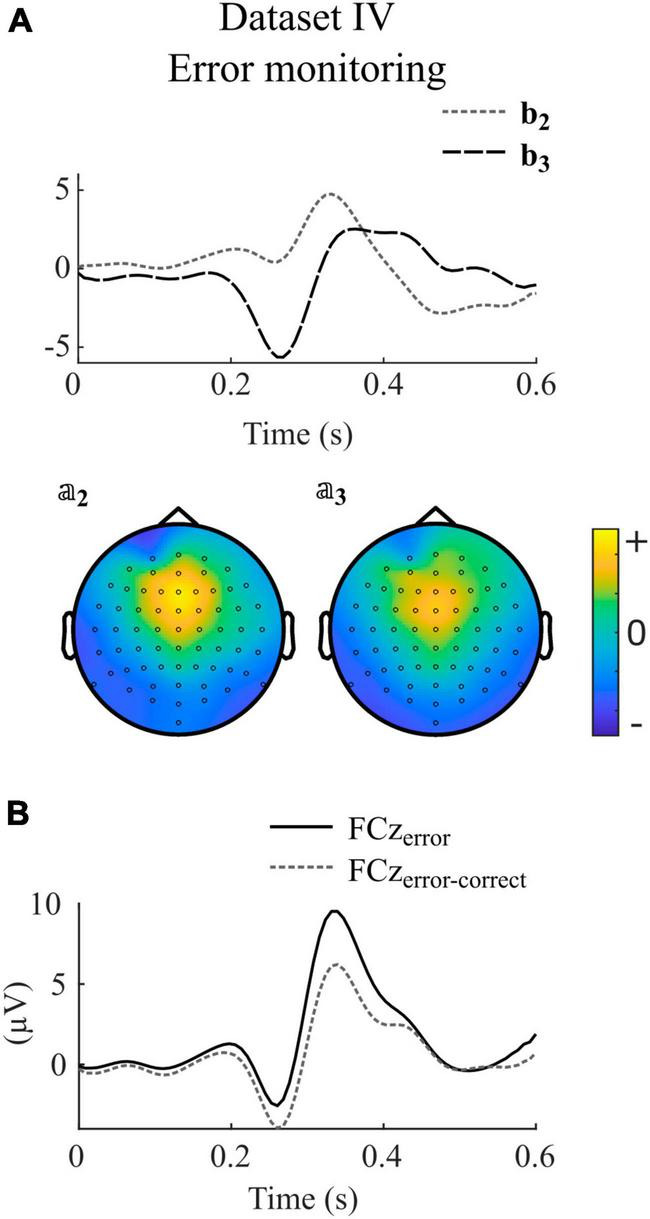
Selected canonical components as obtained with impulse model functions for dataset IV. **(A)** Model signal coefficients (upper panel) and the corresponding activation patterns (second row) of participant *Subject03*, session *S1*. Units of *b_j_* and 𝕒_*j*_ are arbitrary. Positive values in 𝕒_*j*_ indicate similar shape in that channel as in *b_j_*, negative values indicate a reversed shape. **(B)** Average signal and difference wave at electrode FCz. The signals correspond to the same session as shown in **(A)**. The electrode was selected to show the ERPs that are commonly thought to control the BCI.

Finally, Figure 5A shows an example where the second and third components shows properties of error-related negativity, as reflected by *b*_2_, *b*_3_, 𝕒_2_, 𝕒_3_ and activity at the FCz electrode (Figure 5B).

## 4 Discussion

Here we introduced a toolbox for decoding sequences of ERPs. Many BCIs that rely on attention processes use sequences of target and nontarget events to encode different selectable choices and enable a repetition of events to increase the signal-to-noise ratio. The approach used in the toolbox is based on optimal channel weighting using CCA and was combined with classification based on maximum correlation in previous work (Reichert et al., 2015, 2017, 2020a; 2020b; 2022). Optimal channel set selection is a key factor in BCI performance and can be either a hypothesis- (Chavarriaga and Millán, 2010) or data-driven selection (Lal et al., 2004; Ghaemi et al., 2017). Hypothesis-driven selection takes the same channels for each subject, neglecting individual differences. Data-driven channel selection utilizing the classifier repeatedly might be computationally expensive, and the approach might be restricted to the specific task. Another approach is to visually inspect the features of training data, after which an expert decides which channels and time intervals are to be used (Biasiucci et al., 2018). However, this approach is impractical and time consuming. Here we used CCA to extract the components most predictive for the task and used only three of them to serve as surrogate channels, which resulted in decoding accuracies comparable to those reported in the publications that refer to the datasets. The correlation-based feature space was well suited for all four classifier approaches implemented in the toolbox. Using the signals in surrogate channels as the feature space was advantageous for SVM and LDA classifiers, underscoring the high performance of these classifiers in high dimensional feature spaces (Hastie et al., 2009).

We applied average model signals and impulse model signals to estimate the spatial filters. Both resulted in identical spatial filters when only target events were involved for their estimation. For classification, the spatial filters were applied to all data and consequently resulted in identical decoding accuracies for both model signals. In contrast, when nontarget events were involved in the estimation of spatial filters to model a difference wave, in datasets II and III, impulse model signals resulted in higher decoding accuracies compared to average model signals, taking advantage of the arbitrary time course that they can model. This advantage was specifically prominent when the nontarget events evoked ERPs that had similarities with the ERPs of target events. For example, in the VSA dataset, the N2pc is a small modulation of the N2 potential, evoked in response to the visual stimuli, which are identical for both conditions. Similarly, in the RSVP speller dataset, ERPs are evoked after each centrally presented stimulus, but only the attention to the target evokes additional activity. In the matrix speller dataset, we did not find significant differences when considering the difference waves, neither in the spatial filter nor in the decoding accuracies. This indicates that not only the P300 component but also the visual evoked potentials are absent or negligible in non-target events. This supports the finding of Treder and Blankertz (2010), where the accuracy of a matrix speller dropped from almost 100% correct in overt mode to around 40% in covert mode. A similar result was found by Brunner et al. (2010), who demonstrated that the reason for the performance drop in covert mode was the lack of an early response over visual cortex. Altogether, the analysis of the first two datasets corroborates the fact that gaze-dependent matrix spellers are primarily driven by the visual input rather than attention processes.

In all datasets, the average model signals resulted in lower or similar decoding accuracies compared to impulse model signals. Finally, the choice of the model signal, along with other parameters, depends on the experimental protocol. We conclude that the contrast option should be enabled if the driving signal is a modulation of ERPs and can be disabled if the ERP itself is the driving signal. Impulse model signals should be selected, if spatially distinct sources contribute to the discrimination of classes. Average model signals lead to comparable results but have a lower computational load, if the number of channels is lower than the number of time points in the analysis window, and if the ERP itself, rather than its modulation, is relevant. As a classification approach, we would opt for the combination of the canonical component feature space and LDA or SVM, as they showed consistently high performance.

The results achieved with the ERPCCA approach were comparable to the results achieved in the studies referring to the data sets. We have not found studies that have so far also analyzed datasets I-III. For dataset IV, different approaches have achieved improved accuracy compared to the original work and to our results (Rahimi et al., 2020; Yasemin et al., 2023). Recently, convolutional neural networks (CNN) and related techniques have been increasingly used to decode ERPs and were utilized to provide insights into cognitive processing, e.g., Borra et al. (2022, 2023). While some works have shown increased accuracy using CNNs compared to standard techniques (Lawhern et al., 2018; Simões et al., 2020), others argue that no advantage can be found if appropriate feature extraction is performed (Farahat et al., 2019; Vařeka, 2020). However, such deep learning approaches require a high volume of training data and high computational costs, which makes them impractical for BCI use.

The analysis of the four datasets using the ERPCCA toolbox demonstrated that the approach of applying CCA for ERP decoding is effective for various types of brain responses used for BCI control. The benefit compared to existing toolboxes for ERP decoding is that it is not limited to classification of single epochs but is especially suited for decoding sequences of ERPs and that no channel selection is required. The approach is not limited to BCIs but could be applied to any dataset incorporating ERPs of two conditions. Furthermore, it is not applicable only to EEG but could also be used with MEG, electrocorticograms, and related brain signal representations as well. Importantly, the proposed toolbox can be easily used after standard preprocessing, both for offline and online analyses.

## Data availability statement

Publicly available datasets were analyzed in this study. This data can be found here: http://bnci-horizon-2020.eu/database/data-sets and https://doi.org/10.5281/zenodo.8188857.

## Ethics statement

Ethical approval was not required for the study involving humans in accordance with the local legislation and institutional requirements, and since all datasets were taken from publicly accessible online repositories. Written informed consent to participate in this study was not required from the participants or the participants’ legal guardians/next of kin in accordance with the national legislation and the institutional requirements.

## Author contributions

CR: Conceptualization, Formal Analysis, Methodology, Writing – original draft. CS-R: Validation, Writing – review & editing. HH: Project administration, Writing – review & editing. SD: Formal Analysis, Validation, Writing – review & editing.

## Footnotes

1
https://gitlab.com/christoph.reichert/erpcca

2
https://www.cs.cornell.edu/people/tj/svm_light

3
https://github.com/treder/mvpa-light

4
http://bnci-horizon-2020.eu/database/data-sets

5
https://doi.org/10.5281/zenodo.8188857

## Supplementary material

Supplementary Table 1 Average decoding accuracies and standard deviations across subjects for datasets I-IV. The first datasheet shows cross-validation results obtained with different parameter settings using the ERPCCA toolbox. The second datasheet shows online simulation results obtained with the parameters we hypothesized to be conclusive for the dataset.
